# A Dose–Response Relationship of Alcohol Consumption with Risk of Visual Impairment in Korean Adults: The Kangbuk Samsung Health Study

**DOI:** 10.3390/nu14040791

**Published:** 2022-02-14

**Authors:** So Young Han, Yoosoo Chang, Yejin Kim, Chul Young Choi, Seungho Ryu

**Affiliations:** 1 Department of Ophthalmology, Kangbuk Samsung Hospital, School of Medicine, Sungkyunkwan University, Seoul 03181, Korea; acylia@naver.com; 2 Center for Cohort Studies, Total Healthcare Center, Kangbuk Samsung Hospital, School of Medicine, Sungkyunkwan University, Seoul 04514, Korea; yoosoo.chang@gmail.com (Y.C.); reenya@live.co.kr (Y.K.); 3 Department of Occupational and Environmental Medicine, Kangbuk Samsung Hospital, School of Medicine, Sungkyunkwan University, Seoul 04514, Korea; 4 Department of Clinical Research Design & Evaluation, SAIHST, Sungkyunkwan University, Seoul 06351, Korea

**Keywords:** alcohol, cohort studies, visual impairment, vision loss

## Abstract

Visual impairment is a global health problem that leads to poor quality of life. The aim of the study was to examine the dose–response relationship between alcohol consumption and incident visual impairment (VI). This longitudinal cohort study consisted of 287,352 Korean adults who attended health screenings between March 2011 and December 2017 and were followed for up to 8.8 years (median, 4.9 years). Participants were categorized based on their average alcohol consumption. VI was defined as bilateral visual acuity (VA) worse than 0.3 logMAR. We identified 8320 cases of new-onset bilateral VI (incidence rate, 6.0/1000 person-years). Increased alcohol intake was positively and dose-dependently associated with elevated incidence of VI (*p*_trend_ < 0.001). With lifetime abstinence (reference), the multivariable-adjusted hazard ratios (HRs) (95% confidence intervals (CIs)) for incident VI with alcohol intake of <10, 10 to <20, 20–39.9, and ≥40 g/day were 1.07 (0.96–1.19), 1.15 (1.03–1.30), 1.15 (1.01–1.30), and 1.23 (1.08–1.40), respectively. Frequent binge drinking (≥once/per week) was associated with elevated risk of VI (HRs, 1.22; 95% CIs: 1.13–1.32). Former drinkers, particularly men, were at a higher risk for incident VI than lifetime abstainers. Similar associations were observed on evaluating changes in alcohol consumption and other confounders as time-varying covariates. Alcohol consumption, both in moderation and excess, was associated with increased VI incidence.

## 1. Introduction

Visual impairment (VI) is an important public health problem for an aging population [1,2]. VI is associated with low vision-related quality of life, injury, and depression [3,4,5,6]. The exact mechanism of VI is not entirely understood; however, major vision loss causing ocular diseases, such as age-related macular degeneration (AMD) and cataracts, increase with age [7]. Visual acuity (VA) indicates ocular health, and is granted as a “vital sign” of ocular function [8]. Thus, identifying reversible causes of VI can help preserve quality of life and decrease the accompanying health burden.

Alcohol is a well-known modifiable risk factor for the several major diseases worldwide [9]. Long-term periods of alcohol consumption or binge drinking damage multiple organs, mainly causing liver diseases. Alcohol induces oxidative stress that produces reactive oxygen species owing to ethanol metabolism, resulting in the malfunction of tissues and cells [10,11]. Inadequate diet with chronic alcohol access or impaired digestive absorption after alcohol intake results in vitamin deficiencies, which causes problems in various organs, including the eyes [12,13]. Brain impairment induced by alcohol [14] may interrupt the interaction between the eyes and brain as well as damaging the optic nerve or paralyzing eye muscles. Several studies have investigated whether alcohol consumption affects ocular disorders, including the development or progression of cataracts, AMD, and diabetic retinopathy; however, the results are inconsistent [15]. Limited studies have evaluated the relationship between alcohol and VI [16,17,18,19], and their results were conflicting. Moreover, in most previous studies, the definitions of alcohol consumption frequency and history varied greatly and/or the data interpretation were restricted because of cross-sectional design, small sample size, lack of analysis on differential effect by sex, or disregard for differential impact of former and binge drinking.

Therefore, this study aimed to examine the longitudinal association of alcohol consumption and drinking patterns with the development of VI, while evaluating for time-dependent measures of change in alcohol drinking status and other confounders over the follow-up period, in a large cohort of young and middle-aged Korean adults who attended repeated health screenings. To address abstainer bias, we differentiated former drinkers from lifetime abstainers.

## 2. Materials and Methods

### 2.1. Study Population

We used a subsample of the Kangbuk Samsung Health Study, a cohort study of Korean adults who participated in comprehensive annual/biennial health examinations at the clinics of Kangbuk Samsung Hospital Total Healthcare Screening Center in Seoul and Suwon, South Korea [20].

**Figure 1:**
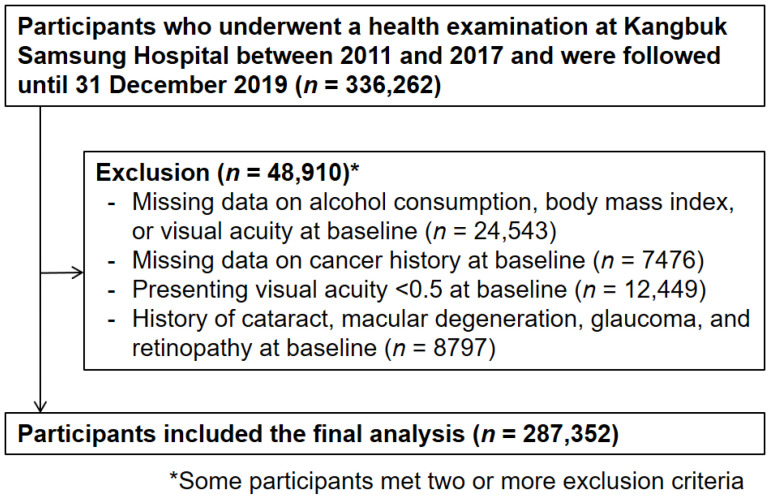
Flowchart for the selection of the study participants.

A total of 336,262 participants who attended health-checkup examinations including vision tests from March 2011 to December 2017, and who underwent at least one follow-up vision test before 31 December 2019, were included in the study (Figure 1). A total of 48,910 participants had ≥one exclusion criterion at the baseline; the final analytic sample included 287,352 participants.

This study was approved by the Institutional Review Board of Kangbuk Samsung Hospital (KBSMC 2020-04-024), and the requirement for written informed consent was waived because the data obtained from routine health screenings were de-identified.

### 2.2. Data Collection

Information on demographic factors, alcohol consumption habits, diet and other health habits, and medical history were assessed at each visit with standardized, self-reported questionnaires [20]. Clinical data regarding ocular disorders, including cataracts, glaucoma, macular degeneration, and other types of retinopathies, were also collected. Physical activity was evaluated using the Korean version of the International Physical Activity Questionnaire Short Form [21,22] into inactive, minimally active, and health-enhancing physically active. [23]. Cardiovascular disease (CVD) was defined as physician-diagnosed heart disease and stroke; cancer was defined as any type of physician-diagnosed malignancy.

Questions regarding alcohol consumption involved the lifetime and current drinking status, drinking frequency, and number of drinks per drinking day, as described previously [24]. Participants who were lifetime abstainers except for a ritual sip during inevitable ceremonies were defined as never-drinkers. Current alcohol use was subdivided as per frequency of alcohol consumption and amount of alcohol consumed per drinking day. The average daily alcohol consumption was estimated using the frequency and amount of alcohol consumed per drinking day. Preferentially, current alcohol consumption of 0, 0.1–<10, 10–<20, 20–<40, and ≥40 g/day was classified as non-drinking, light drinking, moderate drinking, heavy drinking, and heavier drinking, respectively [25]. Thereafter, considering the lifetime abstinence history, the alcohol consumption patterns were classified as lifetime abstinence; current abstinence; and light, moderate, heavy, or heavier drinking. Current abstainers were defined as those who had previously consumed alcohol but were nondrinkers at the visit of their health screening. Participants were also asked about alcohol flushing, which represents aldehyde dehydrogenase 2 (ALDH2) deficiency, assessed using the following inquiry used to classify ALDH2-deficient individuals in South Korea and other East Asian countries [26,27]. We asked, “Do you have a tendency to develop facial flushing immediately after drinking as little as one alcoholic drink?”; participants who answered “yes” were classified as alcohol flushers, while those who answered “no” were classified as alcohol non-flushers.

Binge drinking was evaluated using the third question in the concise Alcohol Use Disorders Identification Test (AUDIT) designed by the World Health Organization (WHO) to screen individuals with harmful alcohol consumption [28], which is “how often do you have six or more drinks on one occasion?,” with the following responses: never, less than monthly, monthly, weekly, and daily or almost daily.

Height, weight, and blood pressure (BP) were determined by trained nurses. Obesity was defined by a body mass index (BMI) ≥ 25 kg/m^2^, which is the cutoff value for diagnosing obesity in Asians [29]. Hypertension was defined as a BP ≥ 140/90 mmHg, history of physician diagnosed hypertension, or present usage of antihypertensive medications.

Fasting blood measurements included the evaluation of glycemic parameters, lipid profiles, and high-sensitivity C-reactive protein (hsCRP) levels. Insulin resistance was estimated with the homeostatic model assessment of insulin resistance (HOMA-IR) equation: fasting blood insulin (μU/mL) × fasting blood glucose (mmol/L)/22.5. Diabetes mellitus was defined as fasting serum glucose ≥126 mg/dL, HbA1c ≥6.5%, or ongoing antidiabetic medication use.

The participants presenting VA while wearing their own spectacles was measured for each eye using Early Treatment Diabetic Retinopathy Study (ETDRS) scales placed at a distance of 3 m [30]. The presenting VA (either VA with naked eye vision or with own spectacle) was evaluated because it reflects real-world VA [31]. VA was recorded as a decimal, which is the general measurement unit in clinics in Korea, and was then converted to the logMAR scale. For the main analysis, VI was defined as VA worse than 0.5 (cutoffs of 0.5; 20/40 Snellen; 0.3 logMAR) in both eyes [18,19,32].

### 2.3. Statistical Analysis

Participant characteristics are presented according to the alcohol consumption categories (mean with SD for continuous variables showing normal distribution, median with interquartile range for skewed continuous variables, and proportion for categorical variables) and were compared using ANOVA for continuous variables or chi-square tests for categorical variables. The distribution of continuous variables was evaluated, and right-skewed variables, including triglycerides, liver enzymes, hsCRP, and HOMA-IR, were log transformed for ANOVA.

The main outcome was the incident binocular VI. Each participant was followed up from their baseline examination until either the occurrence of binocular VI or the last health screening performed before 31 December 2019, whichever occurred first. The incidence rate was calculated as follows: the number of incident VI cases ÷ the number of follow-up person-years. To account for interval censoring a parametric proportional hazards model was used (stpm command in STATA), because if new-onset VI was observed at follow-up, VI must have occurred at an unidentified time point between the visit. [33]. In these models, the baseline hazard function was parameterized with restricted cubic splines in log time with four degrees of freedom.

The hazard ratio (HR) and 95% confidence interval (CI) were estimated for incident VI according to the alcohol consumption category, drinking frequency, number of drinks per drinking day, and frequency of binge drinking. Models were primarily adjusted for age and sex and then further adjusted for center (Seoul or Suwon), year of screening examination, BMI (continuous), physical activity (inactive, minimally active, health-enhancing physically active, or unknown), smoking (never, former, current smoking, or unknown), total energy intake (in quintiles or missing), educational level (high school graduate or less, community college or university graduate, graduate school or more, or unknown), medication for dyslipidemia, and history of CVD, diabetes, and/or hypertension. We conducted additional analyses introducing alcohol drinking status and other confounders as time-varying covariates.

Further analyses were stratified by age (<40 vs. ≥40 years), smoking status (never vs. ≥ ever smoking), health-enhancing physical activity (no vs. yes), BMI (<25 vs. ≥25 kg/m^2^), and flushing (no vs. yes). Interactions between subgroup factors were evaluated using likelihood ratio tests that compared models with vs. without multiplicative interaction terms.

STATA version 16.0 (STATA Corp LP., College Station, TX, USA) was used for all analyses. *p*-values less than 0.05 were considered as statistically significant.

## 3. Results

The average (standard deviation (SD)) age of study participants at baseline was 37.8 (7.8) years, and 57.6% of the participants were men (Table 1). The prevalence of lifetime abstainers and current abstainers (former drinkers) were 3.2% and 8.6%, respectively. Among current drinkers, participants with greater alcohol consumption were more likely to be older, male, have poorer lipid profiles, and have higher levels of BMI, liver enzymes, HOMA-IR, hsCRP, and total energy intake.

During 1,385,492.7 follow-up person-years (median follow-up of 4.9 years; interquartile range, 2.8–6.9 years; maximum 8.8 years), 8320 new-onset VI (incidence rate of 6.0 per 1000 person-years) were identified. Increased alcohol intake categories were positively associated with an increased risk of incident VI in a dose–response manner (*p* for trend <0.001) (Table 2). After adjustment for potential confounders, the multivariable-adjusted HR (95% CIs) for incident VI comparing an alcohol intake of <10, 10 to <20, 20–39.9, and ≥40 g/day with lifetime abstinence as a reference were 1.07 (0.96–1.19), 1.15 (1.03–1.30), 1.15 (1.01–1.30), and 1.23 (1.08–1.40), respectively (*p* for trend <0.001). This pattern showed a stronger tendency in men than in women, but the sex-based interaction was not statistically significant (*p* = 0.051). In men, former drinking was also correlated with a higher risk for incident VI with lifetime abstinence as a reference (HR, 1.69; 95% CI: 1.12–2.55).This association was similarly observed when introducing changes in alcohol consumption and other confounders during follow-up as time-varying covariates.

We also performed analyses while taking into account for medication use which may affect VI. The proportion of antidepressants or psychoactive drug usage was only 1.03%, and the results and tendencies were the same after further adjustment for psychoactive substance use (Supplementary Materials, Table S2).

A higher frequency and number of drinks per drinking day were associated with a higher risk of VI in a dose-dependent manner among men, whereas a high quantity per drinking day but not frequency of drinking was associated with an increased risk of VI in women (Table 3). Frequent binge-drinking was associated with increased risk of VI in a dose-dependent manner in both men and women.

The association between alcohol consumption and VI was consistently observed without significant interaction by predefined subgroups (Supplementary Materials, Table S1).

## 4. Discussion

In the present cohort study of young and middle-aged Korean adults, alcohol consumption was dose-dependently associated with the incidence of VI, and the risk remained significant after adjustment for measured confounders. This association started from a non-heavy drinking level. Notably, low levels of alcohol consumption did not have a protective effect on VI. Former drinking was significantly associated with an increased risk of incident VI, especially among men, supporting the importance of controlling abstainer bias raised by earlier studies. Moreover, binge drinking of weekly frequency or more was consistently associated with a higher risk of VI both in men and women. To the best of our knowledge, our study is by far the largest longitudinal study to examine the association of alcohol consumption with the risk of VI.

Several previous studies have analyzed the effects of alcohol on VI, showing conflicting outcomes. A population-based study of 38,903 French adults reported no association between alcohol consumption and VI prevalence [18]. However, a cross-sectional study of 42,713 middle-aged adults in the United States of America showed that consuming more than one drink/drinking day and binge drinking were positively associated with self-reported VI [16]. A population-based study of 8445 Chinese adults reported that heavy and high-frequency drinkers were associated with significantly higher odds of prevalent VI, whereas moderate drinkers (1–14 drinks per week) had lower odds of prevalent VI than nondrinkers [17]. Opposingly, in a prospective cohort of 9548 participants, those who did not drink over the past year had higher odds of incident VI than did occasional drinkers (at least one serving per week) [19]. Although our findings corroborate previously reported harmful effect of excessive drinking on VI, we did not observe a protective effect of alcohol intake on incident VI at any consumption level. The protective effect of light or moderate alcohol intake documented in prior literature is argued to result from residual confounding related to medical conditions that had led to abstinence when nondrinkers were chosen as the reference group. In fact, the protective association between alcohol consumption and VI in a previous report disappeared after a history of CVD or any ocular condition was considered [16]. In our study of relatively young and healthy adults, an independent relationship of alcohol consumption with incident VI began from non-heavy drinking level, suggesting that, compared to lifetime abstinence, even non-heavy drinking can increase the VI risk.

Our findings show that the association between alcohol intake and VI risk may vary by sex and may be stronger in men than in women. Moreover, men who were current abstainers but former drinkers showed a significantly higher risk of VI; however, this pattern was not observed in women. Though the reasons for the difference observed by sex are not fully understood, sex-based differences between drinking patterns and/or social and physical factors leading to abstinence may partly explain the finding. For instance, several women abstain from alcohol due to pregnancy during their 20s and 30s, and alcohol is not as important to women’s social roles as it is to men’s [34]. Additionally, women are also more susceptible to adverse health effects for a given amount of alcohol than men, partly because the absorption and metabolization of alcohol occurs differently between men and women; women have relatively lesser body water than men with similar body weight, and thus, women may reach higher blood alcohol concentrations after drinking an equivalent amount of alcohol [35,36]. Similarly, the visual deterioration score was significantly higher in women after the intake of the same amount of alcohol (450 mL of wine) in a prospective study of 37 healthy participants in Spain [37]. In the United States of America, alcohol use in men is decreasing, and gaps between the sexes are narrowing. Besides visual problems, women are more susceptible to systemic diseases related to alcohol, such as liver inflammation, cardiovascular diseases, and certain cancers [38]. Such physiological factors may also prevent excessive drinking in women. In Korea, there is a culture of social censure of women’s drinking, and female drinkers have more chances to quit drinking during their lifetime or become mild drinkers. Consequently, some of the female participants in this study may have difficulty maintaining the frequency or amount of drinking they marked on the survey during the follow-up period. Moreover, some female participants may have under-reported drinking [39], possibly leading to misclassification bias. Therefore, further studies are recommended using objective measures of alcohol intake, such as carbohydrate-deficient transferrin or phosphatidyl ethanol. In contrast, drinking is likely to be socially reinforced to a greater extent among men, therefore providing less opportunity for men to quit. Thus, many current male abstainers may have had refrained from drinking due to medical-related issues that could also aggravate VI risk. Another notable finding is that while the frequency of drinking had a significant positive relationship with VI in a dose–response manner in men, the risk of VI in women was not associated with drinking frequency. According to previous literature, men tend to prefer strong alcoholic beverages such as vodka or liquor [40] and are more likely to be involved in heavy or binge drinking [41], whereas women typically prefer mild alcoholic beverages such as beer or wine and are likely to be mild drinkers. This tendency is also reflected in our data wherein 91% of those who consumed ≥40 g/day of alcohol were men. Conclusively, higher overall VI risk observed with an increased frequency of alcohol consumption in men than in women may be related to more frequent binge-drinking episodes in men, suggesting that the frequency of excessive or binge drinking may be a more important determinant than the overall frequency of consumption in explaining the association of alcohol consumption with VI. This is further supported by our finding that a high frequency of binge drinking (≥once a week) was associated with a significantly higher risk of VI both in men and women. Further studies are necessary to confirm or refute the sex-related differences in the association of alcohol consumption and VI and to evaluate whether the association changes after considering types of alcoholic beverages and detailed drinking patterns, which were not available in our dataset.

It is unclear how alcohol increases the VI incidence, but there are several potential explanations. Although still debatable, excessive alcohol consumption increases oxidative stress, which could aggravate cataracts and AMD [15,42]. Case–control prospective studies [43,44] revealed that alcohol administration disturbs tear film, which may lead to ocular surface diseases. Alcohol damages the optic nerve [45] and aggravates hereditary diseases of the optic nerve, causing severe vision loss [46]. Fusion disruption [47] and oculomotor function loss [48,49] were also presented after alcohol consumption. Alcohol loading may adversely affect the rod-cone function in retina [50], causing impairment of color vision [51] and visual discrimination capacity [52]. Moreover, in a prospective population-based study among 11,613 individuals in Copenhagen, heavy drinking increased the risk of arcus cornea, which is one of the most apparent signs of aging, implying that heavy alcohol consumption could accelerate the general aging process [53].

The major strengths of our study include its longitudinal cohort study design, large sample of relatively young and middle-aged men and women, repeated measurements of exposures, and adjustment for confounders including a differentiation between lifetime and current alcohol abstinence, thus enabling us to analyze the independent relationship of the amount alcohol consumption with incident VI while considering the time-varying effect of alcohol consumption and minimizing the abstainer bias observed among nondrinkers.

Our study also has certain limitations. Though presenting VA reflects the real-world vision of daily life [31] and is commonly employed in other studies [31,32,54], best-corrected VA was not provided, and refractive errors were partially corrected with individual’s own spectacles/lenses. We attempted to exclude pre-existing eye diseases; however, the information on eye diseases was based on self-reporting of physician-diagnosed disorders. Additionally, information on dietary factors or all information on medications such as anti-tubercular drugs, antiarrhythmic agents, certain antibiotics [55], or anti-rheumatic medications [56] was not assessed in our study, which could have affected both VI and its interaction with alcohol consumption. Thus, potential bias due to unmeasured confounding factors or residual confounding factors cannot be completely excluded. The information on alcohol consumption was also investigated using a self-reported questionnaire, which may result in misclassification due to recall bias. Different types of alcohol are important because alcohol-related harm may differ by types of alcoholic beverage [57,58]. In Korea, soju and beer are the most common sources of alcohol [59], which is consistent with our data. However, it is difficult to analyze the type of alcohol owing to the Korean drinking culture of consuming “bomb cocktails,” a drinking habit of mixing two or more types of alcohol, which makes it difficult to estimate the effect of individual types of alcoholic beverages. Further research should explore the effects of diverse types of alcoholic drinks. Lastly, our research consists of relatively healthy young or middle-aged, educated Koreans; this generalizability of our findings to other populations may be limited.

## 5. Conclusions

In this study, the VI risk was significantly associated with the amount of alcohol consumption, binge drinking, and frequency of binge drinking. Our findings highlight the importance of refraining from heavy and binge drinking to decrease the risk of VI and reduce the ocular health burden. Further research is needed to elucidate the specific pathogenesis of VI related to alcohol consumption in the general population.

## Figures and Tables

**Table 1:** Baseline characteristics according to lifetime drinking status (*n* = 287,352).

Characteristics	Overall	Lifetime Drinking Status					
		Lifetime Abstainer	Current Abstainer	0 to <10 g/Day	10 to <20 g/Day	20 to <40 g/Day	≥40 g/Day
Number	287,352	9311	24,810	133,807	52,288	37,445	29,691
Age (years) ^a^	37.8 (7.8)	43.9 (10.4)	37.8 (8.1)	36.9 (7.2)	37.5 (7.6)	38.7 (8.0)	39.3 (8.0)
Male (%)	57.6	17.4	29.0	43.4	75.9	85.1	91.3
Current smoker (%)	22.7	3.9	7.0	13.1	29.0	40.8	48.2
HEPA (%)	15.8	15.3	13.8	14.4	16.3	18.3	19.9
High education level (%) ^c^	84.7	73.6	81.2	86.8	86.2	83.9	80.0
Hypertension (%)	9.9	10.8	6.2	6.4	11.0	15.5	19.7
Diabetes (%)	3.3	4.2	2.6	2.2	3.4	4.8	6.2
History of CVD (%)	0.8	1.4	0.8	0.6	0.9	1.0	1.3
Medication for dyslipidemia (%)	1.9	4.6	1.7	1.4	1.9	2.4	2.9
Obesity (%) ^d^	28.4	19.9	19.7	21.5	33.7	39.7	45.9
Body mass index (kg/m^2^) ^a^	23.3 (3.4)	22.4 (3.3)	22.4 (3.4)	22.7 (3.3)	23.9 (3.2)	24.4 (3.2)	24.9 (3.2)
Systolic BP (mmHg) ^a^	109.4 (13.0)	107.1 (13.3)	104.2 (12.5)	106.6 (12.3)	111.8 (12.3)	114.4 (12.3)	116.4 (12.3)
Diastolic BP (mmHg) ^a^	70.1 (9.9)	67.9 (9.3)	66.8 (9.3)	68.0 (9.2)	71.7 (9.6)	73.8 (9.9)	75.5 (10.0)
Glucose (mg/dL) ^a^	94.8 (13.9)	94.0 (13.1)	92.2 (12.9)	93.0 (12.0)	95.6 (14.1)	97.8 (15.9)	99.8 (17.5)
Total cholesterol (mg/dL) ^a^	193.3 (34.0)	193.6 (34.9)	189.4 (33.5)	189.9 (33.2)	195.6 (33.9)	198.6 (34.3)	201.1 (35.1)
LDL-C (mg/dL) ^a^	120.2 (32.0)	120.4 (32.5)	114.4 (31.0)	117.3 (31.2)	123.5 (32.2)	125.1 (32.4)	125.8 (32.8)
HDL-C (mg/dL) ^a^	58.8 (15.4)	61.0 (15.2)	60.8 (15.2)	60.3 (15.4)	56.8 (15.1)	56.5 (15.1)	56.6 (14.9)
Triglycerides (mg/dL) ^b^	90 (64–135)	80 (59–114)	77 (57–110)	80 (59–116)	99 (69–147)	112 (77–164)	123 (85–182)
AST (U/L) ^b^	19 (16–24)	19 (16–23)	18 (15–22)	18 (16–22)	20 (17–25)	21 (18–27)	23 (19–29)
ALT (U/L) ^b^	18 (13–28)	15 (12–22)	15 (11–22)	16 (12–24)	20 (14–31)	22 (16–33)	24 (17–36)
GGT (U/L) ^b^	20 (13–35)	14 (11–21)	15 (11–22)	16 (12–25)	25 (16–40)	32 (21–54)	42 (26–71)
hsCRP (mg/L) ^b^	0.4 (0.2–0.9)	0.4 (0.2–0.8)	0.4 (0.2–0.9)	0.4 (0.2–0.8)	0.5 (0.2–1.0)	0.5 (0.3–1.0)	0.5 (0.3–1.0)
HOMA-IR ^b^	1.21 (0.80–1.80)	1.16 (0.76–1.73)	1.14 (0.76–1.67)	1.16 (0.77–1.73)	1.25 (0.82–1.86)	1.29 (0.85–1.93)	1.34 (0.88–2.02)
Total energy intake (kcal/day) ^b,e^	1523.5 (1157.9–1924.9)	1446.0 (1096.4–1838.1)	1464.0 (1091.8–1867.0)	1480.3 (1119.1–1870.8)	1548.8 (1198.3–1947.4)	1594.7 (1235.9–2007.8)	1659.6 (1285.1–2095.5)

Data are expressed as ^a^ mean (standard deviation), ^b^ median (interquartile range), or percentage. ^c^ ≥college graduate; ^d^ body mass index ≥25 kg/m^2^. ^e^ among 202,998 participants with reliable estimated energy intake levels (within three standard deviations of the log-transformed mean energy intake). ALT, alanine aminotransferase; AST, aspartate aminotransferase; BP, blood pressure; CVD, cardiovascular disease; GGT, gamma-glutamyl transpeptidase; HDL-C, high-density lipoprotein cholesterol; HEPA, health-enhancing physically active; hsCRP, high-sensitivity C-reactive protein; HOMA-IR, homeostasis model assessment of insulin resistance; LDL-C, low-density lipoprotein cholesterol. SI conversion factors: To convert glucose to millimoles per liter, multiply by 0.0555; total cholesterol, HDL-C, and LDL-C to millimoles per liter, multiply by 0.0259; triglycerides to millimoles per liter, multiply by 0.0113; AST, ALT, and GGT to microkatals per liter, multiply by 0.0167; and hsCRP to nanomoles per liter, multiply by 9.524.

**Table 2:** Hazard ratios (95% CI) for visual impairment by the category of alcohol consumption based on lifetime drinking history and current drinking status (*n* = 287,352).

Alcohol Consumption Category	Person-Years (PY)	Incident Cases	Incidence Density (10^3^ PY)	Age & Sex Adjusted HRs (95% CI)	Multivariable-Adjusted HR (95% CI) ^a^	HR (95% CI) ^b^ in Model Using Time-Dependent Variables
Total (*n* = 287,352)						
Lifetime abstainer	40,090.3	412	10.3	1.00 (reference)	1.00 (reference)	1.00 (reference)
0.1 to <10 g/day	639,831.4	3909	6.1	1.08 (0.97–1.19)	1.07 (0.96–1.19)	1.09 (0.98–1.21)
10 to <20 g/day	256,412.5	1359	5.3	1.18 (1.05–1.32)	1.15 (1.03–1.30)	1.19 (1.06–1.34)
20 to <40 g/day	182,329.7	957	5.2	1.19 (1.05–1.35)	1.15 (1.01–1.30)	1.21 (1.07–1.38)
≥40 g/day	140,300.0	781	5.6	1.29 (1.13–1.47)	1.23 (1.08–1.40)	1.31 (1.15–1.49)
*p* for trend ^c^				<0.001	<0.001	<0.001
Current abstainer	126,528.8	902	7.1	1.09 (0.97–1.23)	1.09 (0.97–1.23)	1.17 (1.04–1.32)
Women (*n* = 121,819)						
Lifetime abstainer	32,802.1	386	11.8	1.00 (reference)	1.00 (reference)	1.00 (reference)
0.1 to <10 g/day	352,293.3	2756	7.8	1.05 (0.94–1.17)	1.05 (0.94–1.17)	1.07 (0.96–1.19)
10 to <20 g/day	57,145.8	486	8.5	1.20 (1.04–1.37)	1.17 (1.02–1.34)	1.22 (1.06–1.40)
20 to <40 g/day	24,653.1	214	8.7	1.24 (1.04–1.47)	1.19 (1.004–1.41)	1.29 (1.08–1.53)
≥40 g/day	11,400.7	101	8.9	1.32 (1.06–1.64)	1.24 (0.99–1.55)	1.30 (1.03–1.64)
*p* for trend ^c^				<0.001	0.003	<0.001
Current abstainer	88,359.0	702	7.9	1.02 (0.90–1.16)	1.03 (0.90–1.16)	1.12 (0.98–1.27)
Men (*n* = 165,533)						
Lifetime abstainer	7288.1	26	3.6	1.00 (reference)	1.00 (reference)	1.00 (reference)
0.1 to <10 g/day	287,538.1	1153	4.0	1.42 (0.96–2.10)	1.40 (0.95–2.07)	1.32 (0.91–1.92)
10 to <20 g/day	199,266.7	873	4.4	1.51 (1.02–2.24)	1.48 (1.00–2.18)	1.40 (0.96–2.05)
20 to <40 g/day	157,676.6	743	4.7	1.53 (1.04–2.27)	1.47 (0.99–2.18)	1.43 (0.98–2.08)
≥40 g/day	128,899.3	680	5.3	1.68 (1.13–2.48)	1.59 (1.07–2.36)	1.56 (1.07–2.29)
*p* for trend ^c^				<0.001	0.005	<0.001
Current abstainer	38,169.7	200	5.2	1.70 (1.13–2.56)	1.69 (1.12–2.55)	1.59 (1.07–2.37)

The *p* value for the interaction of sex and smoking status for risk of bilateral visual impairment was 0.051 (multivariable model). ^a^ Estimated from parametric proportional hazard models. Multivariable model was adjusted for age, sex (only for total), center, year of screening exam, BMI, physical activity, smoking, total energy intake, educational level, medication for dyslipidemia, history of CVD, history of diabetes, and history of hypertension. ^b^ Estimated from parametric proportional hazard models with alcohol consumption category, physical activity, smoking, total energy intake, BMI, medication for dyslipidemia, history of diabetes, history of hypertension, and history of CVD as time-dependent categorical variables and for baseline age, sex, center, year of screening exam, and education. ^c^ After excluding current abstainer. BMI, body mass index; BP, blood pressure; CI, confidence interval; HR, hazards ratio; CVD, cardiovascular disease; HOMA-IR, Homeostasis Model Assessment of Insulin Resistance; hsCRP, high sensitivity C-reactive protein.

**Table 3:** Hazard ratios (95% CI) for visual impairment by drinking pattern after excluding former drinkers (*n* = 262,542).

Drinking Pattern	Multivariable-Adjusted HR (95% CI) ^a^		
	Total	Women	Men
Frequency of drinking (drinks/week)			
0	1.00 (reference)	1.00 (reference)	1.00 (reference)
1–2	1.09 (0.98–1.21)	1.07 (0.95–1.19)	**1.49 (1.002–2.21)**
3–4	1.18 (1.04–1.33)	1.13 (0.96–1.32)	**1.62 (1.09–2.41)**
5–6	1.18 (0.98–1.41)	1.02 (0.74–1.42)	**1.67 (1.09–2.56)**
7	1.34 (0.97–1.85)	1.31 (0.75–2.27)	**1.83 (1.06–3.13)**
*p* for trend	0.002	0.173	0.004
Number of drinks consumed per drinking day			
0	1.00 (reference)	1.00 (reference)	1.00 (reference)
1–2	1.06 (0.95–1.18)	1.02 (0.91–1.14)	1.49 (0.99–2.23)
3–5	1.11 (1.00–1.25)	1.09 (0.97–1.23)	1.43 (0.96–2.13)
≥6	1.27 (1.13–1.43)	**1.33 (1.16–1.53)**	**1.61 (1.08–2.39)**
*p* for trend	<0.001	<0.001	0.003
Frequency of binge drinking ^b^			
Never	1.00 (reference)	1.00 (reference)	1.00 (reference)
<Once a month	1.06 (0.99–1.14)	1.08 (0.99–1.18)	0.99 (0.87–1.13)
Once a month	1.09 (1.004–1.19)	**1.15 (1.02–1.30)**	1.02 (0.89–1.15)
≥Once a week	1.22 (1.13–1.32)	**1.25 (1.10–1.42)**	**1.15 (1.03–1.29)**
*p* for trend	<0.001	<0.001	0.001

^a^ Estimated from parametric proportional hazard models. Multivariable model was adjusted for age, sex (only for total), center, year of screening exam, BMI, physical activity, smoking, total energy intake, educational level, medication for dyslipidemia, history of CVD, history of diabetes and history of hypertension. ^b^ Among 219,602 participants with available binge drinking data after excluding former drinkers. CI, confidence interval; CVD, cardiovascular disease; HR, hazard ratio. Boldface emphasizes main findings.

## Data Availability

The data are not available to be shared publicly because we do not have permission from the Institutional Review Board to distribute the data. However, data are available from the Kangbuk Samsung Health Study upon request, whose authors may be contacted through the corresponding authors for this manuscript.

## Supplementary Materials

The following supporting information can be downloaded at: https://www.mdpi.com/article/10.3390/nu14040791/s1, Table S1: Hazard ratios (95% CI) for visual impairment according to lifetime drinking status in clinically relevant subgroups. Table S2: Hazard ratios (95% CI) for visual impairment according to lifetime drinking status in clinically relevant subgroups.
