# Supplementary material for: A Dose–Response Relationship of Alcohol Consumption with Risk of Visual Impairment in Korean Adults: The Kangbuk Samsung Health Study
Source: Nutrients. 2022 Feb 14;14(4):791. doi: 10.3390/nu14040791 (PMC8875794; doi:10.3390/nu14040791)
Supplement: Supplementary file 1 [file nutrients-14-00791-s001.zip › nutrients-1572002-supplementary.pdf]

Table S1. Hazard ratios (95% CI) for visual impairment according to lifetime drinking status in clinically relevant subgroups.

Subgroup	Lifetime drinking status						*p* for interaction
	Lifetime abstainer	Current abstainer	0 to <10 g/day	10 to <20 g/day	20 to <40 g/day	≥40 g/day	
**Age (years)**							0.429
<40 (*n* = 189,518)	reference	0.97 (0.78–1.21)	0.96 (0.78–1.18)	1.04 (0.84–1.29)	1.07 (0.86–1.35)	1.07 (0.84–1.35)	
≥40 (*n* = 97,834)	reference	0.97 (0.84–1.12)	0.93 (0.82–1.04)	0.94 (0.82–1.08)	0.91 (0.78–1.05)	1.00 (0.86–1.16)	
Sex							0.051
Women (*n* = 121,819)	reference	0.76 (0.67–0.86)	0.74 (0.66–0.82)	0.77 (0.67–0.88)	0.77 (0.65–0.91)	0.76 (0.61–0.94)	
Men (*n* = 165,533)	reference	1.45 (0.96–2.18)	1.14 (0.77–1.68)	1.20 (0.81–1.77)	1.23 (0.83–1.82)	1.33 (0.90–1.97)	
Smoking							0.564
Never (*n* = 141,285)	reference	1.04 (0.90–1.20)	1.04 (0.92–1.18)	1.10 (0.95–1.27)	1.22 (1.02–1.45)	1.17 (0.94–1.46)	
Ever (*n* = 125,657)	reference	1.33 (0.91–1.94)	1.22 (0.85–1.75)	1.32 (0.92–1.89)	1.28 (0.89–1.84)	1.41 (0.98–2.03)	
HEPA							0.939
No (*n* = 239,293)	reference	1.09 (0.95–1.24)	1.06 (0.94–1.19)	1.13 (0.99–1.29)	1.14 (1.00–1.31)	1.22 (1.05–1.40)	
Yes (*n* = 44,862)	reference	1.08 (0.80–1.45)	1.13 (0.88–1.45)	1.25 (0.96–1.64)	1.18 (0.89–1.56)	1.33 (1.00–1.77)	
BMI (kg/m^2)							0.581
<25 (*n* = 205,697)	reference	1.10 (0.96–1.25)	1.08 (0.96–1.21)	1.19 (1.04–1.36)	1.18 (1.03–1.36)	1.25 (1.08–1.45)	
≥25 (*n* = 81,655)	reference	1.08 (0.83–1.41)	1.06 (0.84–1.33)	1.04 (0.82–1.32)	1.05 (0.82–1.34)	1.15 (0.90–1.47)	
Flushing							0.869
No (*n* = 188,965)	reference	1.09 (0.73–1.63)	1.13 (0.77–1.67)	1.23 (0.83–1.83)	1.21 (0.82–1.80)	1.33 (0.90–1.98)	
Yes (*n* = 79,041)	reference	0.98 (0.73–1.32)	0.96 (0.73–1.26)	1.02 (0.76–1.37)	1.11 (0.81–1.52)	1.09 (0.77–1.54)	

Estimated from parametric proportional hazard models. Multivariable model was adjusted for age, sex (only for total), center, year of screening exam, body mass index (BMI), physical activity, smoking, total energy intake, educational level, medication for dyslipidemia, history of CVD, history of diabetes, and history of hypertension CI, confidence interval; CVD, cardiovascular disease; HR, hazard ratio; HEPA, health-enhancing physically active; high-sensitivity C-reactive protein. SI conversion factors: To convert hsCRP to nanomoles per liter, multiply by 9.524.

Table S2. Hazard ratios (95% CI) for visual impairment by drinking pattern after further adjustment for usage of anti-depressants or psychoactive drugs (*n* = 287,352).

Drinking pattern	Multivariable-adjusted HR (95% CI)[a]		
	Total	Women	Men
Lifetime abstainer	1.00 (reference)	1.00 (reference)	1.00 (reference)
0.1 to <10 g/day	1.07 (0.96–1.19)	1.05 (0.94–1.17)	1.40 (0.95–2.07)
10 to <20 g/day	1.15 (1.03–1.30)	1.17 (1.02–1.34)	1.48 (0.99–2.18)
20 to <40 g/day	1.15 (1.01–1.30)	1.19 (1.003–1.41)	1.47 (0.99–2.18)
≥40 g/day	1.23 (1.08–1.40)	1.24 (0.99–1.55)	1.59 (1.07–2.36)
p for trend	<0.001	<0.001	0.001
Current abstainer	1.09 (0.97–1.23)	1.03 (0.90–1.16)	1.69 (1.12–2.55)

[a]Estimated from parametric proportional hazard models. Multivariable model was adjusted for age, sex (only for total), center, year of screening exam, BMI, physical activity, smoking, total energy intake, educational level, medication for dyslipidemia, history of CVD, history of diabetes, history of hypertension and use of anti-depressants or psychoactive drugs.
